# Supplementary material for: A Cellular Assay for the Identification and Characterization of Connexin Gap Junction Modulators
Source: Int J Mol Sci. 2021 Jan 31;22(3):1417. doi: 10.3390/ijms22031417 (PMC7866863; doi:10.3390/ijms22031417)
Supplement: Supplementary file 1 [file ijms-22-01417-s001.pdf]

International Journal of *Molecular Sciences*

MDPI

Supporting Information

A Cellular Assay for the Identification and Characterization of Connexin Gap Junction Modulators

Azeem Danish[1#], Robin Gedschold[1#], Sonja Hinz[1], Anke C. Schiedel[1], Dominik Thimm[1], Peter Bedner[2], Christian Steinhäuser[2*] and Christa E. Müller[1*]

[1] PharmaCenter Bonn, Pharmaceutical Institute, Pharmaceutical & Medicinal Chemistry, Rheinische Friedrich-Wilhelms-Universität Bonn, An der Immenburg 4, D-53121 Bonn, Germany; christa.mueller@uni-bonn.de

[2] Institute of Cellular Neuroscience, Medical Faculty, Rheinische Friedrich-Wilhelms-Universität Bonn, Venusberg-Campus 1, D-53127 Bonn, Germany

* Correspondence: christa.mueller@uni-bonn.de; Christian.Steinhaeuser@ukbonn.de

#equal contribution

Table of Contents

Figure S1. Expression profile of connexin 43 in wild-type and transfected cell lines 2

Figure S2. Cytotoxicity assay of carbenoxolone 2

Figure S3. Screening results using the developed assay 3

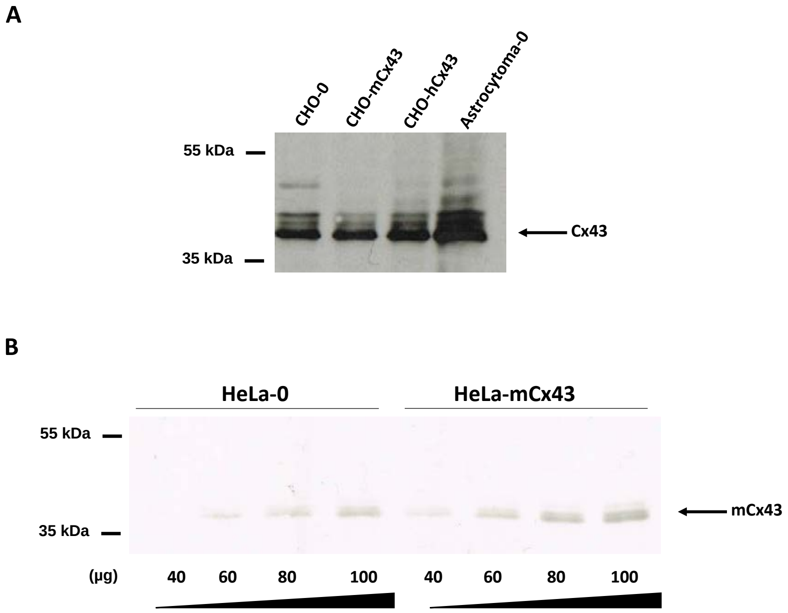

Figure S1. Expression profile of Cx43 in wild-type and Cx43-transfected cell lines. (**A**) Western blot analysis of heterologous expression of human and mouse Cx43 in stable CHO cell lines and endogenous expression of Cx43 in native CHO and astrocytoma cell lines. For the analyses, 30 µg of each protein sample per well was loaded onto SDS-gel. (**B**) Western blot analysis of endogenous Cx43 expression in native HeLa cell lines and heterologous expression of mouse Cx43 in HeLa cells post 48 h transient transfection. Cells were lysed, cleared by centrifugation, and the supernatants were subsequently used for analysis. The Cx43 protein bands were detected using primary polyclonal rabbit anti-Cx43 antibodies (1:2500) and secondary horseradish peroxidase (HRP) conjugated anti-rabbit antibodies (1:4000). Chemoluminescence from nitrocellulose blots was captured on X-ray films which were developed in fixing solutions.

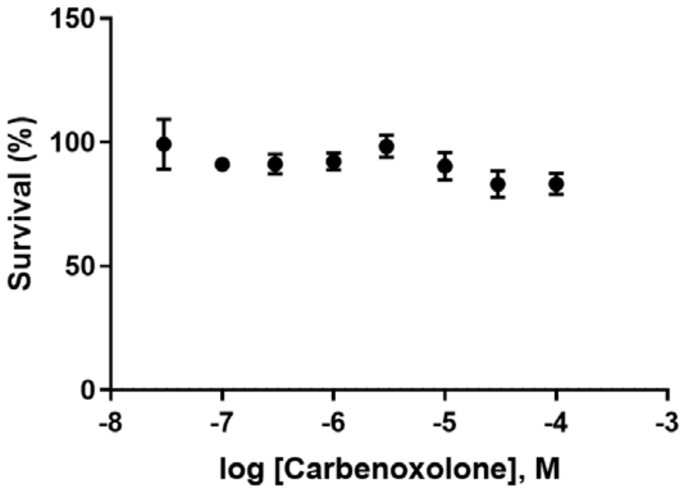

Figure S2. Cytotoxicity of carbenoxolone in HeLa cells measured by the MTT assay. Normalized results (Buffer = 100%; DMSO (20%) = 0%). Data points are means ± SEM of three independent experiments in duplicates.

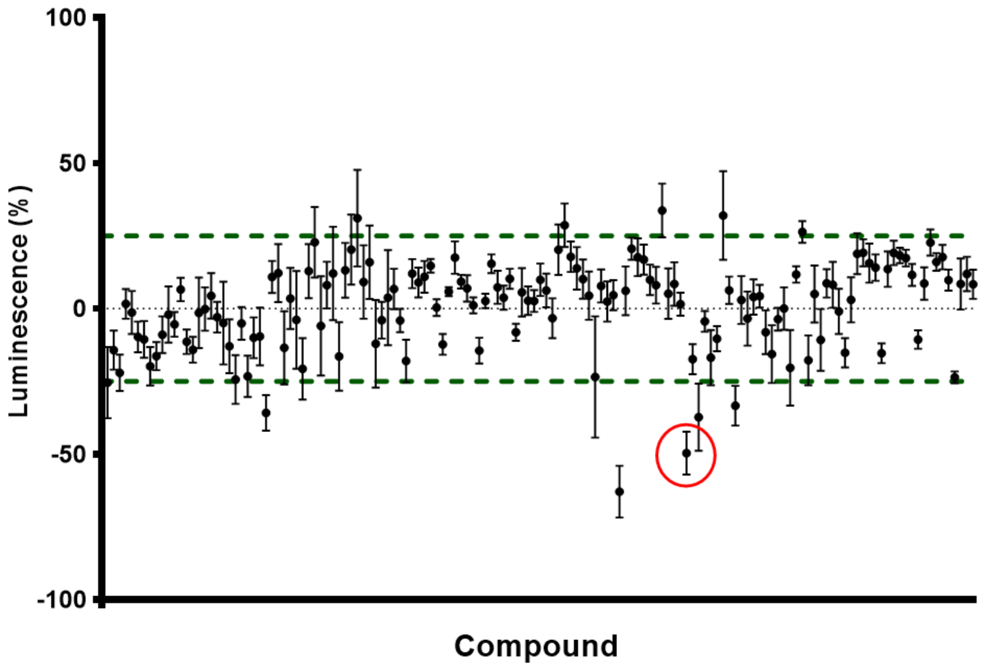

Figure S3. Screening results. A library comprising 143 small bioactive molecules was screened by the new GJ assay at a concentration of 10 μM. Data points represent means ± SEM of three independent experiments in duplicates. Compounds with >25% change in luminescence compared to the signal induced by CGS-21680 were classified as hits. CGS-21680 effect = 0%; Buffer = -100%. Circled in red is U-54494A hydrochloride which was identified as a confirmed hit compound.
